# Most Pleiotropic Effects of Gene Knockouts Are Evolutionarily Transient in Yeasts

**DOI:** 10.1093/molbev/msae189

**Published:** 2024-09-06

**Authors:** Li Liu, Yao Liu, Lulu Min, Zhenzhen Zhou, Xingxing He, YunHan Xie, Waifang Cao, Shuyun Deng, Xiaoju Lin, Xionglei He, Xiaoshu Chen

**Affiliations:** Department of Immunology and Microbiology, Zhongshan School of Medicine, Sun Yat-sen University, Guangzhou, China; MOE Key Laboratory of Gene Function and Regulation, State Key Laboratory of Biocontrol, Innovation Center for Evolutionary Synthetic Biology, School of Life Sciences, Sun Yat-sen University, Guangzhou, China; Department of Immunology and Microbiology, Zhongshan School of Medicine, Sun Yat-sen University, Guangzhou, China; Department of Immunology and Microbiology, Zhongshan School of Medicine, Sun Yat-sen University, Guangzhou, China; Department of Immunology and Microbiology, Zhongshan School of Medicine, Sun Yat-sen University, Guangzhou, China; Department of Immunology and Microbiology, Zhongshan School of Medicine, Sun Yat-sen University, Guangzhou, China; MOE Key Laboratory of Gene Function and Regulation, State Key Laboratory of Biocontrol, Innovation Center for Evolutionary Synthetic Biology, School of Life Sciences, Sun Yat-sen University, Guangzhou, China; Evolutionary Ecology, Ludwig-Maximilians-Universität München, Planegg-Martinsried, Germany; Department of Immunology and Microbiology, Zhongshan School of Medicine, Sun Yat-sen University, Guangzhou, China; Department of Immunology and Microbiology, Zhongshan School of Medicine, Sun Yat-sen University, Guangzhou, China; Department of Immunology and Microbiology, Zhongshan School of Medicine, Sun Yat-sen University, Guangzhou, China; MOE Key Laboratory of Gene Function and Regulation, State Key Laboratory of Biocontrol, Innovation Center for Evolutionary Synthetic Biology, School of Life Sciences, Sun Yat-sen University, Guangzhou, China; Department of Immunology and Microbiology, Zhongshan School of Medicine, Sun Yat-sen University, Guangzhou, China; Key Laboratory of Tropical Disease Control, Ministry of Education, Sun Yat-sen University, Guangzhou, China

**Keywords:** pleiotropic, evolution, transcription factors, transcriptomes, morphological traits, eQTLs

## Abstract

Pleiotropy, the phenomenon in which a single gene influences multiple traits, is a fundamental concept in genetics. However, the evolutionary mechanisms underlying pleiotropy require further investigation. In this study, we conducted parallel gene knockouts targeting 100 transcription factors in 2 strains of *Saccharomyces cerevisiae*. We systematically examined and quantified the pleiotropic effects of these knockouts on gene expression levels for each transcription factor. Our results showed that the knockout of a single gene generally affected the expression levels of multiple genes in both strains, indicating various degrees of pleiotropic effects. Strikingly, the pleiotropic effects of the knockouts change rapidly between strains in different genetic backgrounds, and ∼85% of them were nonconserved. Further analysis revealed that the conserved effects tended to be functionally associated with the deleted transcription factors, while the nonconserved effects appeared to be more ad hoc responses. In addition, we measured 184 yeast cell morphological traits in these knockouts and found consistent patterns. In order to investigate the evolutionary processes underlying pleiotropy, we examined the pleiotropic effects of standing genetic variations in a population consisting of ∼1,000 hybrid progenies of the 2 strains. We observed that newly evolved expression quantitative trait loci impacted the expression of a greater number of genes than did old expression quantitative trait loci, suggesting that natural selection is gradually eliminating maladaptive or slightly deleterious pleiotropic responses. Overall, our results show that, although being prevalent for new mutations, the majority of pleiotropic effects observed are evolutionarily transient, which explains how evolution proceeds despite complicated pleiotropic effects.

## Introduction

The classic view that a gene often affects only a few, if not a single, traits can be traced back to the pea experiments conducted by Gregor Mendel (Stearns 2010). This view has been a cornerstone of genetics, and much research has focused on making connections between genes and specific traits (Stenseth et al. 2022; Botstein and Risch 2003). Numerous examples can be found where genes are named after the traits they influence. For instance, the HTT gene is linked to Huntington's disease (Walker 2007), and the FOXP2 gene is involved in the development of speech and language (Lai et al. 2003; Lai et al. 2001).

However, it is crucial to recognize that many genes have pleiotropic effects, meaning they can influence multiple traits or have broader impacts on biological processes (Stearns 2010; Paaby and Rockman 2013; Nagy et al. 2018). A classic example is the gene for phenylketonuria (PKU). It is caused by a deficiency of an enzyme called phenylalanine hydroxylase, which converts the essential amino acid phenylalanine to tyrosine. Due to a defect in the single gene that codes for this enzyme, PKU results in a variety of phenotypes, including mental retardation, eczema, and pigmentation problems (Paul 2000). Similarly, the gene for cystic fibrosis was initially believed to primarily affect the lungs and digestive system but has since been found to have a more extensive range of effects on other organs and systems in the body (Li et al. 2014). As research has advanced, it has become increasingly apparent that genetic systems are complex and interconnected, and the relationship between genes and traits is often more nuanced than previously believed (Hill and Zhang 2012; Wagner and Zhang 2011; Solovieff et al. 2013).

Recent large-scale mapping of gene knockout or knockdown effects in several model organisms suggests that the classic view likely represents the exception rather than the norm. Specifically, among the 767 yeast knockout mutants under 21 environmental conditions, ∼30% were highly pleiotropic, with growth defects observed in as many as 14 conditions (Dudley et al. 2005). In a previously described screening study, comprehensive assessments of phenotypes encompassing developmental, biochemical, physiological, and organ functions were performed in mice, in which 449 genes had been knocked out (de Angelis et al. 2015). In total, 2,947 phenotype annotations were ascribed to 320 genes, and 65% of these genes had more than 1 phenotypic hit. Moreover, an analysis of variants in 257 protein-coding genes revealed their association with more than 1 human disease, accounting for 12% of all disease-causing proteins reported in UniProt (Ittisoponpisan et al. 2017). These empirical findings indicate that gene pleiotropy is widespread and robust in nature.

Because pleiotropy constrains evolutionary adaptation, a puzzling issue is how such extensive gene pleiotropy originated and evolved (Stearns 2010; Guillaume and Otto 2012; Pavličev 2016). In our previous study, we examined various knockout effects, including those on gene expression and morphological differences resulting from the absence of *HAP4*, a transcription factor (TF) in yeast (Liu et al. 2020). Our findings revealed that a large proportion of the effects lacked heritability and exhibited poor conservation in closely related strains, implying that pleiotropic effects may not hold equivalent evolutionary importance. Interestingly, a recent study on the growth of 3,786 knockout mutants of 4 *Saccharomyces cerevisiae* strains reported that only 9% to 24% of deletion phenotypes were conserved within species, indicating that evolutionarily unstable gene knockout effects may be prevalent (Galardini et al. 2019). However, this study primarily focused on growth rates under different conditions, which was not independent and does not suggest pleiotropy. Overall, there is a lack of comprehensive and rigorous studies on pleiotropy.

Here, we conducted a comprehensive investigation on the knockout effects of TFs to reveal the evolutionary characteristics of pleiotropy. We knocked out 100 TFs, which represented a broad range of TF types, in 2 strains of *S. cerevisiae*, BY and RM. Biological replicates were performed for each TF knockout, and we subsequently measured gene expression and examined cell morphology to determine the pleiotropic effects of each knockout in both strains. We scrutinized the effects on gene expression and cell morphology within species and discovered that the conservation of pleiotropic effects of the knockout mutations was very low. Since gene knockout can be taken as a “new” null mutation, we hypothesized that the cause of this instability may be traced back to the adaptive process of a mutation. That is, whenever a new mutation occurs, it may disturb the normal balance within the cell in an unfamiliar manner, leading to various responses (Galhardo et al. 2007; Hill et al. 2021; Zhang et al. 2022). Natural selection therein preserves adaptive responses while gradually eliminating maladaptive or slightly deleterious pleiotropic responses. To test this hypothesis, we further examined the pleiotropic effects of standing genetic variations in a population consisting of approximately 1,000 haploid segregants produced from a cross of BY and RM. Our results conclusively indicated that the pleiotropy of older polymorphic sites was lower than that of younger sites, strongly suggesting that pleiotropy may decline with evolution.

## Results

### TF Knockouts Generate Various Pleiotropic Effects on Gene Expression

**Fig. 1.**
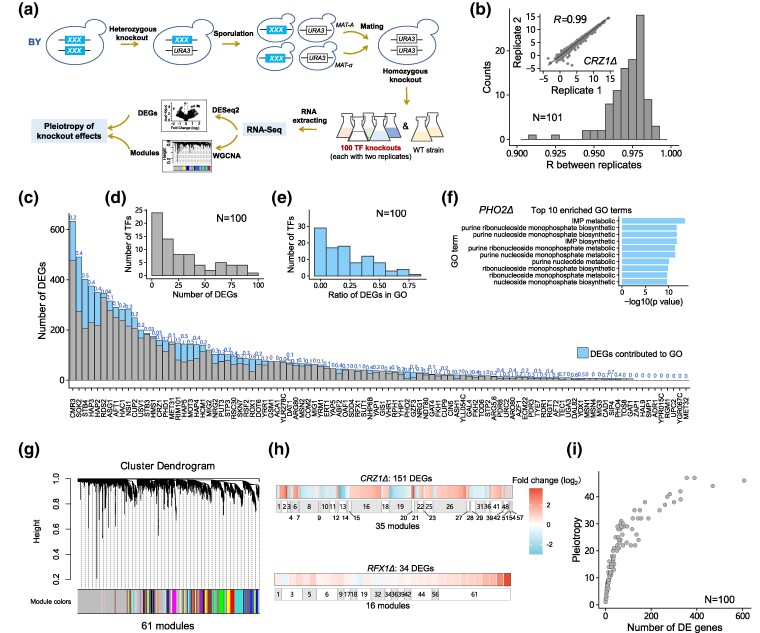
Knockouts of 100 TFs generate various pleiotropic effects on gene expression in BY. a) The experimental process of homozygous knockouts and transcriptome measurement in BY. b) The distribution of Pearson's *R* of expression profiles between 2 biological replicates for knockouts of 100 TFs and the wild-type strain of BY, and the inset represents the case of *PHD1Δ*. c) Numbers of DEGs for 100 TF knockouts in BY. The blue color indicates the fractions of DEGs contributing to enriched GO terms, and the values are labeled. d) The distribution of the number of DEGs across 100 TFs. e) The distribution of the ratio of DEGs contributing to enriched GO terms. f) The top 10 enriched GO terms of *PHO2Δ*. g) The cluster dendrogram of coexpressed modules estimated by WGCNA. h) The deletion of *CRZ1* resulted in 151 DEGs that were dispersed in 35 coexpressed modules, and the deletion of *RFX1* resulted in 34 DEGs that were dispersed in 16 coexpressed modules. The module IDs are labeled in the panel below. i) The number of DEGs and corresponding pleiotropic effects estimated by the coexpressed modules across 100 TF knockouts in BY.

To generate homozygous knockouts for 100 TFs in the diploid BY strain, we started by replacing 1 copy of the target gene with a *URA3* cassette through homologous recombination. Subsequently, we induced sporulation in the heterozygous knockout and conducted crosses between 2 segregants, each carrying null alleles at the target gene locus but with different mating types. This procedure allowed us to successfully obtain the desired homozygous knockout (Fig. 1a; see Materials and Methods). Of the 100 TFs, 42 function as activators, 13 function as repressors, 3 play dual roles in regulation, and the functions of the remaining TFs are currently unclear based on the Saccharomyces Genome Database (SGD) (Cherry et al. 1998) (supplementary table S1, Supplementary Material online). Since TFs directly regulate the spectra and levels of gene expression, we first examined the effects of these knockouts on gene expression. The transcriptomes of 2 biological replicates for each knockout and the wild-type strain were examined (supplementary tables S2 and S3, Supplementary Material online), and the performances of 2 replicates within each group were highly consistent (Fig. 1b). The effects of these TFs on gene expression were evaluated by comparing the transcriptome in each knockout to that in the wild-type strain by DESeq2 (Love et al. 2014).

In the BY strain, the number of differentially expressed genes (DEGs) in the knockouts of 100 TFs varied from 1 to 633, with a median of 37 at a stringent threshold of an expression change (up- or downregulated) of at least 1.2-fold (FC) and strong statistical significance (*P*_adj_ < 0.01) (Fig. 1c and d; supplementary fig. S1a and table S4, Supplementary Material online; see Materials and Methods). Gene Ontology (GO) enrichment analysis for DEGs of each TF was performed and GO terms that were functionally related to the focal TF were derived for 75 TFs, and the ratio of DEGs contributing to these GO terms varied from 0.03 to 0.8 (Fig. 1e). For example, *PHO2Δ* regulates genes involved in phosphate metabolism, and its deletion resulted in significant changes in the expression of 30 genes. Among these genes, 24 were clustered in dozens of GO terms annotated as phosphate biosynthetic and metabolic processes (Fig. 1f). This observation was consistent with the results of a previous study (Liu et al. 2020), suggesting the prevailing pleiotropy of TF null mutations. Most of the remaining 25 TFs have relatively few DEGs and therefore no enriched GO terms, with some minor exceptions associated with special environments such as glucose depletion–related MIG1 and MIG2 (Lutfiyya and Johnston 1996).

Due to their coregulation among many genes, the number of DEGs cannot provide an accurate estimation of the pleiotropic effects of gene knockouts. To effectively quantify pleiotropy, we followed a previous approach (Hämälä et al. 2020) to cluster the expression levels of all genes across 100 knockouts using weighted gene coexpression network analysis (WGCNA) (Langfelder and Horvath 2008). Genes that failed to be assigned to modules were not considered. In total, 4,597 genes were clustered into 61 coexpressed modules (Fig. 1g; see Materials and Methods). The number of modules in which DEGs dispersed was taken as the pleiotropy value of each TF knockout on gene expression. For example, 151 DEGs for *CRZ1Δ* were dispersed in 35 modules, indicating a pleiotropy value of 35 for *CRZ1Δ* (Fig. 1h). It was clear that these DEGs were not dispersed randomly in 35 modules but were enriched in specific modules, including 8, 10, 16, 19, 26, and 41, echoing the internal connections of these genes. For *RFX1Δ*, ∼30% of DEGs (10/34) were enriched in module 61 (Fig. 1h). In summary, the pleiotropy values estimated by the coexpressed modules varied from 1 to 47, with a median of 16 across 100 TFs (Fig. 1i). While this method may favor nonconserved changes, the conclusion of pervasive pleiotropic effects is compatible with that derived from DEGs.

### Weak Conservation of Pleiotropic Effects of TF Knockouts on Gene Expression

**Fig. 2.**
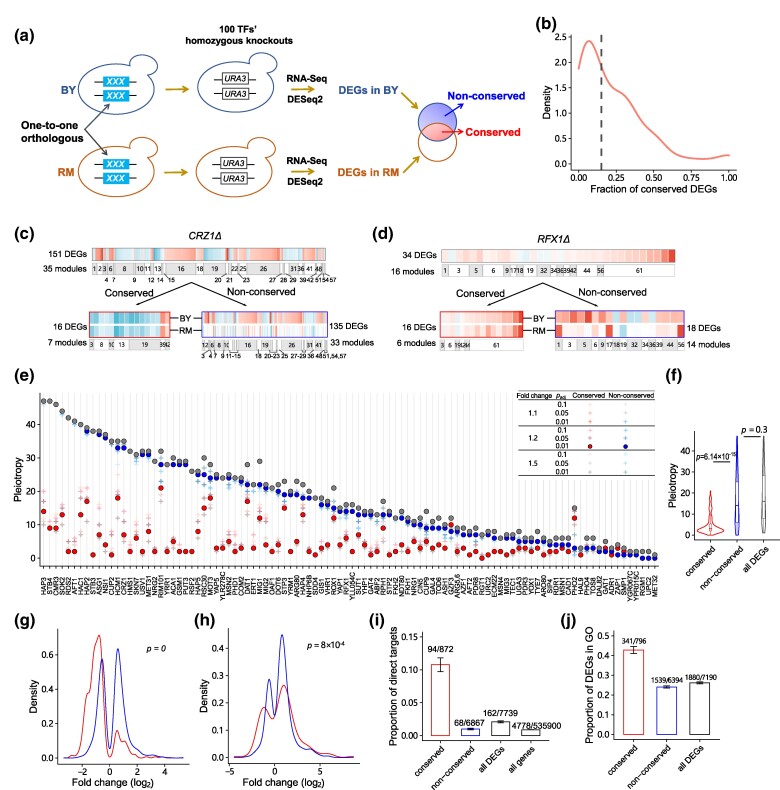
Most pleiotropic effects on gene expression are nonconserved within species. a) A diagram defining conserved and nonconserved DEGs. b) The density curve of the fraction of conserved DEGs across 100 TF knockouts in BY. The dashed line indicates the median value. c) Conservation of pleiotropy for the *CRZ1* knockout. d) Conservation of pleiotropy for the *RFX1* knockout. e) The pleiotropic effects of 100 TF knockouts were categorized into conserved or nonconserved via a comparison with the DEGs identified in RM. Conserved pleiotropic effects are colored red, nonconserved pleiotropic effects are colored blue, and overall pleiotropic effects are colored gray. Specific thresholds used for DEG identification in RM are listed in the top-right corner. FC indicates both up- and downregulation. f) The comparison of conserved, nonconserved, and overall pleiotropic effects. The *P* values obtained from the Wilcoxon test between each pair of groups are labeled. g) The density curves of log_2_FC for conserved and nonconserved effects in 42 activators. The *P* values obtained from the Kolmogorov–Smirnov test between the 2 curves are labeled. h) The density curves of log_2_FC for conserved and nonconserved effects in 13 repressors. The *P* values obtained from the Kolmogorov–Smirnov test between the 2 curves are labeled. i) Proportions of direct targets of TFs in different categories. Error bars represent the SE. j) Proportions of DEGs contributing to enriched GO terms in different categories. Error bars represent the SE.

We aimed to determine whether the pleiotropic effects of TF knockouts on gene expression were conserved within species. To explore this issue, we constructed homozygous knockouts of 1-to-1 orthologs of 100 TFs in another *S. cerevisiae* strain (RM) using the same experimental process described above (Fig. 2a). These 2 strains, BY and RM, differ by approximately 0.5% at the genomic level. We examined the transcriptomes of 2 biological replicates for each knockout and wild-type strain of RM (supplementary table S5 and figs. S1 and S2, Supplementary Material online) and defined DEGs for each TF in RM at the same statistical threshold (supplementary table S4, Supplementary Material online).

It was striking that the fractions of DEGs in BY conserved in RM were generally low across 100 TF knockouts, with a median fraction of 15% for conserved DEGs (Fig. 2b; supplementary table S6, Supplementary Material online). We then examined pleiotropy by visualizing the coexpressed modules (those containing both conserved and nonconserved effects). For instance, in *CRZ1Δ*, 16 of the 151 DEGs were conserved in the RM knockout, and these 16 DEGs were dispersed in 7 coexpressed modules, indicating that the pleiotropy value of this gene decreased from 35 to 7. On the other hand, the pleiotropy value of the 135 nonconserved DEGs was 33, which was comparable to the overall pleiotropy value (Fig. 2c). Similarly, for *RFX1Δ*, 47% of DEGs were conserved in RM, while the pleiotropy values for conserved and nonconserved effects were 6 and 14, respectively (Fig. 2d).

For the 100 TF knockouts, we observed notable differences in the pleiotropy values of conserved and nonconserved effects. The pleiotropy values of conserved effects ranged from 0 to 21, with a median of 3. In contrast, the pleiotropy values of nonconserved effects showed a wider range, spanning from 0 to 47, with a median value of 14 (Fig. 2e). We observed a significantly decrease in the pleiotropy values of conserved effects (Fig. 2f; Wilcoxon test: *P* = 6.14 × 10^−15^), while those of nonconserved effects remained relatively stable and did not significantly differ from the overall pleiotropy value (Fig. 2f; Wilcoxon test: *P* = 0.3). These findings suggest that nonconserved effects contribute significantly to the overall pleiotropy of knockout mutations.

We also investigated the influence of statistical thresholds on the detection of DEGs in the RM strains. Our comprehensive investigation in which diverse FC and *P*_adj_ thresholds were used revealed that certain portions of conserved and nonconserved effects were influenced by the specific thresholds utilized (Fig. 2e inset; supplementary fig. S3, Supplementary Material online). However, we observed that the choice of statistical thresholds had little effect on the overall pattern of our findings, i.e. the pleiotropy value of nonconserved effects was higher than that of conserved effects. The classification of coexpressed modules by WGCNA would affect the clustering of DEGs, thus altering the pleiotropy value. We investigated the conservation of pleiotropic effects of the knockouts using coexpressed modules generated by different parameters and found that the above pattern was consistent (supplementary fig. S4, Supplementary Material online).

The next question we aimed to address was “What causes different levels of conservation?” There was a total of 7,739 DEG-TF pairs in BY. Based on their performances in RM, they were assigned to either the conserved (872) or nonconserved (6867) category. Since the direction of expression change directly reflects the regulatory mechanism of TFs, the FC distributions were examined in 42 activators and 13 repressors. Regarding the knockout effects of activators, we observed that the downregulation of DEGs was particularly pronounced in the conserved category, while nonconserved category showed no significant upregulated or downregulated tendency (Fig. 2g). Conversely, regarding the knockout effects of repressors, the conserved category showed a different signal, with upregulated expression being dominant (Fig. 2h). This pattern held true even after the removal of the top 3 genes (*RIB4*, *MDH2*, and *AAP1*) whose expression levels were most frequently affected by more than 70 TFs (supplementary fig. S5, Supplementary Material online). These observations suggest that conserved effects may be closely connected to focal TFs, while nonconserved effects are not.

To confirm this finding, we extracted the targets of TFs from YEASTRACT (Teixeira et al. 2023). Of the 7,739 DEG-TF pairs, only 2.1% (162/7739) were verified as targets of the corresponding TF. Notably, 10.8% (94/872) of the genes in the conserved category were targets of focal TFs. In comparison, those in the nonconserved category accounted for just 1% (68/6867), which was comparable to the value in the genomic background of 0.9% (4778/535900) (Fig. 2i; χ*^2^* test: *P* = 0.4). These findings indicate that the verified targets were significantly enriched in the conserved effects. We also examined the number of DEGs contributing to enriched GO terms in both categories. DEGs in the conserved category showed an approximately 2-fold enrichment compared to those in the nonconserved category (Fig. 2j), providing further support that genes in the conserved category are more directly affected by focal TFs.

### Weak Conservation of Pleiotropic Effects of Gene Knockouts on Cell Morphology

**Fig. 3.**
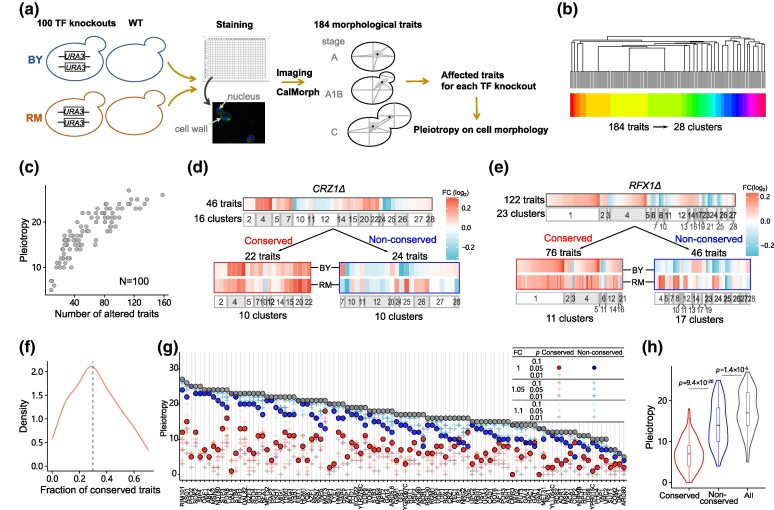
Most pleiotropic effects on cell morphology are nonconserved within species. a) The experimental process of measuring morphological traits for 100 TF knockouts in BY and RM. b) The clustering tree of 184 morphological traits of 100 TF knockouts. Different colors indicate different clusters. c) The number of altered traits and corresponding pleiotropic effects estimated by trait clusters across 100 TF knockouts in BY. d) The deletion of *CRZ1* resulted in 46 traits that were significantly altered and dispersed in 16 clusters. Among them, 22 traits were conserved, and 24 were nonconserved. e) The deletion of *RFX1* resulted in 122 traits that were significantly altered and dispersed in 23 clusters. Among them, 76 traits were conserved, and 46 were nonconserved. f) The density curve of the fraction of conserved traits across 100 TF knockouts in BY. The dashed line indicates the median value. g) The pleiotropic effects of 100 TF knockouts were categorized into conserved or nonconserved via a comparison with the significantly altered traits identified in RM. Conserved pleiotropic effects are colored red, nonconserved pleiotropic effects are colored blue, and overall pleiotropic effects are colored gray. Specific thresholds used for significantly altered traits identification in RM are listed in the top-right corner. FC indicates both up- and downregulation. h) The comparison of conserved, nonconserved, and overall pleiotropic effects. The *P* values obtained from the Wilcoxon test between each pair of groups are labeled.

Changes in gene expression levels do not necessarily correspond to changes in cell morphologies, since morphologies are determined by many biochemical processes, whose changes might be compensated by one another. We directly investigated the pleiotropic effects of 100 TF knockouts on cell morphology in BY and RM (Fig. 3a). To quantify these effects, we harvested yeast cells at the same growth state and stained them with 2 fluorescent dyes to label the cell wall and nucleus (see Materials and Methods). Using high-resolution imaging of single cells, we quantified 184 cell morphological traits for each strain, with each trait value calculated from >300 cells (supplementary table S7, Supplementary Material online). These traits represent the stage, size, shape, and localization of the mother cell and bud, as previously described (Ohya et al. 2005; Okada et al. 2015). Each knockout was performed in triplicate, with 3 technical replicates for each biological replicate. We observed strong correlations between replicates, indicating the stability of measurements (supplementary fig. S6, Supplementary Material online).

Similar to gene expression, most cell morphological traits are correlated with each other. We then grouped the 184 cell morphological traits into 28 clusters using affinity propagation clustering based on all trait values (Bodenhofer et al. 2011) (Fig. 3b; see Materials and Methods). To analyze the significance of variation for each trait in each TF knockout in each strain, we used a linear mixed-effects model (Bates et al. 2015) that included all replicates of the TF knockouts and wild-type strains. Traits with *P* < 0.05 are defined as altered traits. As with gene expression, we first examined the effects on cell morphology in BY and found that 9 to 158 traits were significantly altered across 100 TFs (supplementary table S8, Supplementary Material online). We then used the number of clusters in which altered traits were dispersed to estimate the pleiotropic effects of each TF on cell morphology. The pleiotropy values ranged from 5 to 27, with a median of 17 for 100 TF knockouts in BY (Fig. 3c). When *CRZ1Δ* and *RFX1Δ* were used as examples, *CRZ1Δ* resulted in 46 significantly altered morphological traits that dispersed into 16 trait clusters, resulting in a pleiotropy value of 16 (Fig. 3d). For *RFX1Δ*, the pleiotropy value on cell morphology was 23, corresponding to 122 significantly altered traits (Fig. 3e).

We then analyzed the conservation of altered traits. Only 47.8% (22/46) of altered traits associated with *CRZ1Δ* were significantly altered in the RM strain, while 62.3% (76/122) of altered traits associated with *RFX1Δ* were. The median fraction of conserved altered traits was 29.8% for 100 TFs (Fig. 3f, supplementary fig. S7 and table S9, Supplementary Material online). Consistent with our observations on gene expression, the pleiotropy value of conserved effects on cell morphology was relatively low, while the pleiotropy value of nonconserved effects was similar to the overall pleiotropy value (Fig. 3g and h). The pattern was not sensitive to the statistical thresholds of FC and *P* (Fig. 3g inset). These results suggest that most pleiotropic effects of knockouts on cell morphology are also evolutionarily transient.

### Reduced Pleiotropic Effects of Older Polymorphic Sites

Our study of gene knockouts and their impact on gene expression and cell morphology revealed that null mutations can have various effects, with most of the pleiotropic effects resulting from the null mutation being nonconserved. This led us to speculate about the possibility of a new null mutation that could cause extensive pleiotropic effects in specific genetic backgrounds. Such a mutation could disrupt the cellular balance in an unfamiliar way, resulting in a diverse range of responses, whereas the progress of adaptive evolution, these adverse reactions are gradually eliminated by natural selection. To explore this idea further, we examined the pleiotropic effects of standing genetic variations in a population. A direct inference was that a younger quantitative trait locus (QTL) should affect more traits than an older QTL that had already undergone evolution.

**Fig. 4.**
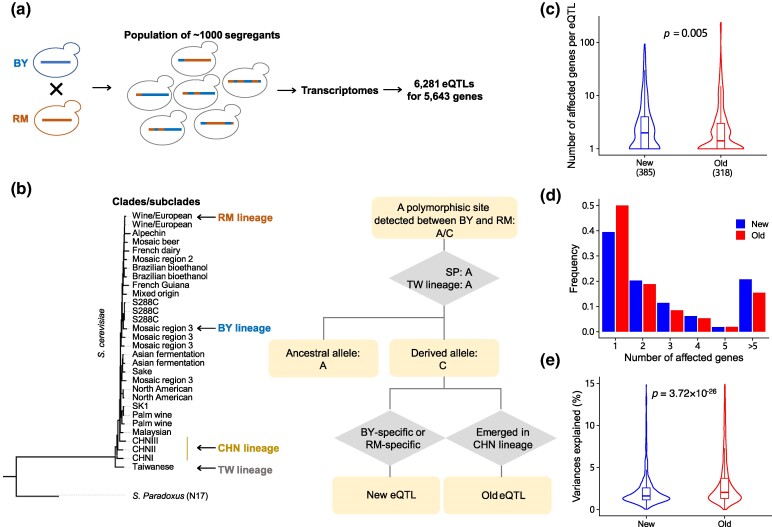
New QTLs affect more traits than old QTLs. a) A diagram of how the segregant panel is constructed. b) The phylogenetic tree showing the Chinese origin of *S. cerevisiae* and the procedure to define new QTLs and old QTLs. The clades of strains for the BY lineage, RM lineage, CHN lineage, and TW lineage are indicated by arrows. c) Comparison of the number of affected genes for new eQTLs and old eQTLs. The *P* values obtained from the Wilcoxon test are labeled. d) Histogram of the number of affected genes for new eQTLs and old eQTLs. e) Comparison of the expression variances explained for new eQTLs and old eQTLs. The *P* values obtained from the Wilcoxon test are labeled.

A panel of ∼1,000 haploid segregants was produced from a cross of BY and RM as in a previous study (Bloom et al. 2013) (Fig. 4a). The expression profiles and genotypes of the segregants were characterized, which led to the discovery of 6,281 expression QTLs (eQTLs) for 5,643 genes (Albert et al. 2018). Each eQTL was analyzed to determine the number of affected genes (supplementary table S10, Supplementary Material online) (Albert et al. 2018). To define the age of these eQTLs, we used the knowledge from a comprehensive study of 1,011 *S. cerevisiae* isolates. It was found that the *S. cerevisiae* originated from the Taiwanese lineage, followed by the Chinese lineage, and then diverged into 26 main lineages due to factors such as geography, environmental niche, and human association (Peter et al. 2018). We defined 2 strains for the BY lineage, 2 strains for the RM lineage, 5 strains for the Chinese lineage, and 3 strains for the Taiwanese lineage, together with 21 randomly sampled strains from other clades for comparison (supplementary table S11, Supplementary Material online). *Saccharomyces paradoxus* (SP), the species most closely related to the *S. cerevisiae* clade, was used as an outgroup. The allele states of eQTLs in various *S. cerevisiae* strains and the SP strain were determined (see Materials and Methods).

To define new and old eQTLs, we only considered those whose allele states in BY or RM were consistent with Taiwanese strains and SP, thereby avoiding SC-specific alleles potentially introduced by recombination instead of mutation (Mancera et al. 2008). We then defined an eQTL with a derived allele that emerges in the Chinese lineage as an “old eQTL.” Similarly, an eQTL with a derived allele that is presented only in BY or RM is defined as a “new eQTL.” For example, if a polymorphic site is found between BY and RM, with alleles A/C, and the alleles in 3 Taiwanese strains and SP were A, then A is the ancestral allele, and C is the derived allele. If the derived allele C is only present in 3 BY strains (i.e. BY specific), then the eQTL with this polymorphic site is considered new. Similarly, the eQTL whose derived allele is RM specific is also defined as a new eQTL. If the derived allele C is found in Chinese strains, then the eQTL is classified as old (Fig. 4b).

We obtained 385 new eQTLs and 318 old eQTLs by that standard, and a noticeable difference between the 2 sets was revealed. The number of genes affected by new eQTLs was significantly higher than that affected by older eQTLs (Fig. 4c; Wilcoxon test: *P* = 0.005). In fact, approximately 50% or 159 of 318 old eQTLs affected the expression of only a single gene (Fig. 4d). On the other hand, 60.5% or 233 of 385 new eQTLs impacted the expression of 2 or more genes (Fig. 4d). Additionally, we analyzed the phenotypic variances explained by the eQTLs in each group and found that old eQTLs contributed more expression variance than new eQTLs (Fig. 4e; Wilcoxon test: *P* = 3.72 × 10^−26^). These findings suggest that new QTLs are more pleiotropic but have smaller effective sizes than old QTLs.

## Discussion

In this study, we individually knocked out 100 TFs from 2 *S. cerevisiae* strains, BY and RM, and investigated their phenotypic consequences on gene expression and cell morphology. We observed pervasive pleiotropic effects of null TF mutations in BY, which were mostly nonconserved in RM. This result is not biased in BY, because the abundant pleiotropic effects found in RM were similarly nonconserved (supplementary figs. S8 and S9, Supplementary Material online). Our results imply that most pleiotropic effects change rapidly in different genetic backgrounds and may therefore have limited evolutionary significance. Further corroborating our theory, in a panel of segregants produced from a cross of BY and RM, we found that compared to evolutionarily new eQTLs, old eQTLs affect fewer genes but have stronger effects. These findings suggest an evolutionary null model of pleiotropy, in which high degrees of pleiotropy accompanying new null mutations rapidly decline over time, rendering most pleiotropic effects evolutionarily transient.

We found a pervasive presence of pleiotropy that can be verified not only by using individual DEGs and cell morphological traits but also by using coexpressed gene modules and trait clusters. Consistent with our findings, a single nucleotide variation within a pleiotropic gene can trigger a cascade of phenotypic alterations that can be conserved within or between species (Hill et al. 2021). For instance, in *Drosophila santomea*, a cis-regulatory substitution in the enhancer of the scute gene leads to additional leg sex comb sensory teeth and the loss of 2 genitalia sensory bristles compared to *Drosophila yakuba* (Nagy et al. 2018). Similarly, variation in the expression of GATA leads to differences in both compound eye size and head shape in 2 closely related *Drosophila* species (Buchberger et al. 2021). Please note that our research focuses on TFs, which are theoretically more pleiotropic than most genes. More investigation into the pleiotropy of non-TFs is needed. Nevertheless, recent large-scale mapping of gene knockouts in several model organisms suggests that nonpleiotropic genes, such as Mendelian genes, may represent the exception rather than the norm.

It has previously been suggested by preliminary evidence that most pleiotropic effects are not conserved (Liu et al. 2020; Galardini et al. 2019). Our study provided unprecedented support for this claim through a combination of (i) novel experimental data derived from multiple genetic backgrounds and phenotypes, (ii) quality experimental data that are highly consistent across replicates and between different genetic backgrounds (no significant difference in the number of DEGs between BY and RM, paired samples *t*-test, *P* = 0.52), and (iii) robust conclusions irrespective of the changes in analytical parameters. Notably, recent studies have underscored the importance of genetic background in the context of mutations (Galardini et al. 2019; Mullis et al. 2018; Adamus-Bialek et al. 2018; Chandler et al. 2013). Higher-order genetic interactions between a mutation and multiple loci can result in significantly different phenotypic effects across backgrounds (Nguyen Ba et al. 2022; Hou et al. 2018). Considering our findings and those from other reports regarding the nonconservative nature of pleiotropy in different genetic backgrounds, we are compelled to contemplate the evolutionary implications of pleiotropy.

To this end, we hypothesized that pleiotropy is rapidly lost during evolution and its low conservation might be a consequence of the rapid loss and gain of pleiotropic functions in each genetic background following divergence. Compatible with this hypothesis, we found that old eQTLs exhibit lower levels of pleiotropy than new eQTLs, suggesting that pleiotropy has declined over time due to natural selection. We should point out that the observed low pleiotropy of old eQTLs could be explained by the decreasing pleiotropy of existing alleles over time or by the shorter survival time associated with pleiotropy alleles. In any case, both explanations emphasize the general disadvantages of excessive pleiotropy. The only difference between them is whether natural selection is more effective in eliminating pleiotropy or in removing pleiotropic alleles. Similar to natural selection against specific mutations versus overall mutation rates, the answer to the above question will depend on the disadvantages of the specific pleiotropic effects versus the overall pleiotropic effects.

In our previous research, we proposed categorizing knockout effects into evolutionarily selected effects and evolutionarily ad hoc effects (Liu et al. 2020). The former supports the biochemical understanding of the gene of interest, while the latter contributes to pleiotropy. Our current study, with its extensive data, bolsters this proposal. We found that conserved effects are more likely to be functionally related, while the nonconserved effects are not. Most pleiotropic effects of gene knockouts are evolutionarily transient, which requires a reconsideration of the biological importance of most knockout effects.

From the perspective of evolutionary genetics, apart from the widespread belief that pleiotropy constrains evolution, how evolution and pleiotropy are shaped by each other remains largely elusive (Stearns 2010; Guillaume and Otto 2012; Pavličev 2016). Based on the findings of this study, it appears that any new null mutation in a population carries the potential to have multiple pleiotropic effects, most of which are deleterious whereas some could be beneficial. Natural selection prunes the deleterious pleiotropic effects over time, resulting in a declining degree of pleiotropy, which facilitates adaptive evolution. Our results indicate that the interaction between pleiotropy and evolution is bidirectional, so that the limitations of pleiotropy on evolution may be alleviated to some extent.

In conclusion, our study provides a new perspective on the origin and evolution of pleiotropy by focusing on its conservation. We believe that understanding the conservatism of pleiotropy can elucidate the complex nature of genetic interactions and their evolutionary implications.

## Materials and Methods

### Selection of TFs

Two hundred TFs correlated with high and medium expert confidence in *S. cerevisiae* were selected from the reference (De Boer and Hughes 2012). Essential yeast genes (Winzeler et al. 1999) were then removed from these TFs. Next, the 1-to-1 orthologs of TFs coexisting in 2 *S. cerevisiae* strains, *BY*4743 (BY) and *RM*11 (RM), were retained (Scannell et al. 2011). Finally, all the remaining 141 TFs in each of these 2 diploid strains were selected for subsequent experiments. The reference genome and corresponding genome annotation of BY and RM were obtained from the SGD (Cherry et al. 1998) version 64-2 and Saccharomyces Genome Resequencing Project version 2 (Liti et al. 2009), respectively.

### Heterozygous Knockouts

Following the yeast transformation protocol (Gietz and Schiestl 2007) with some adjustments, the *URA3* cassette of plasmid pYES2 was used to replace 1 allele of each of the above TFs in BY and RM by a pair of homologous recombination primers that can amplify *URA3* and contain the front and back ∼60 bp sequences of the cDNA encoded by each TF (supplementary table S12, Supplementary Material online). Specifically, the cells of each strain were cultured at 30 °C in 5 mL of yeast peptone dextrose (YPD, 1% yeast extract, 2% peptone, and 2% glucose) overnight until saturation. Then, the cells were diluted to an OD660 of 0.2 and grown for approximately 4 h until the OD660 reached 0.7. Each culture was harvested by centrifugation at 2000 × *g* for 5 min, and then, competent cells were prepared using 0.1 M lithium acetate (LiAc). Subsequently, 240 μL of 50% w/v polyethylene glycol, 30 μL of 1 M LiAc, 10 μL of 10 mg/mL salmon sperm vector DNA, 5 µg of DNA product expanded by homologous recombination primers, and 50 μL competent cells were added to a tube and vortexed for 1 min. The mixture was heat-shocked at 42 °C for 30 min. After washing once in water, each mixture was spread onto *s*ynthetic complete medium plates without uracil (SC-ura) and cultured for 2 to 3 d.

Transformants of individual colonies were selected and confirmed by polymerase chain reaction for correct replacement. Two pairs of transformation-checking primers were designed for each TF (supplementary table S12, Supplementary Material online). The first pair of transformation-checking primers was designed such that one matched within the native TF gene and the other matched upstream of the TF cDNA. The second pair of transformation-checking primers was designed such that one matched within the *URA3* cassette, and the other matched upstream of the TF cDNA. Finally, 3 colonies representing heterozygous knockouts of 121 TF were obtained in both BY and RM.

### Homozygous Knockouts

First, the heterozygous knockouts were made into spores using potassium acetate (KAc) (Elrod et al. 2009). For BY, the liquid method was used for sporulation. Specifically, cells were cultured in 5 mL YPD overnight until saturation. After washing twice with water, 5 mL of 1% KAc was added to the culture and incubated at 25 °C for 5 to 7 d. For RM, the solid method was used for sporulation. Specifically, cells were streaked in YPD plates with 1% KAc and incubated at 25 °C for 5 to 7 d. The spores were collected into tubes, and 500 μL of Y1 buffer (1 M sorbitol and 0.1 M EDTA, pH 7.4), 1 μL of 8 U/μL lyticase, and 0.5 μL of β-mercaptoethanol were added. The mixtures were vortexed for 30 s and incubated at 30 °C for 37 min and at 55 °C for 30 min. The spores were washed once with water, harvested by centrifugation at 12,000 rpm for 1 min, and resuspended in 100 μL mineral oil and 500 μL water. Spores were then collected by centrifugation at 5800 rpm for 10 min. Next, 100 μL of an oil layer, 100 μL of gelatin, and 100 μL of water were added, and the samples were spread onto SC-ura plates at 30 °C for 2 d. Single colonies were selected for amplification using transformation-checking primers to further confirm whether the TFs had been replaced. In addition, the MATa and MATα primers were used to determine their mating types (Astell et al. 1981) (supplementary table S12, Supplementary Material online).

Next, spores of the paired mating types were mixed into 5 mL YPD to form homozygous knockouts. Specifically, BY cells were shaken at 250 rpm for 16 h at 30 °C. For RM, the samples were shaken at 250 rpm for 16 h at 33 °C and left at 30 °C for 1 h. One microliter of culture was then streaked directly onto SC-ura plates for 2 d. Single colonies were selected for amplification, and transformation-checking primers were used to confirm whether the 2 alleles of each TF had been replaced. Furthermore, MATa and MATα primers were used to determine whether they formed diploid strains. Finally, 3 colonies of homozygous knockouts of each of the 100 TFs were obtained in both the BY and RM strains (supplementary table S1, Supplementary Material online), which covers 50% of the TFs with high and medium expert confidence from the reference (De Boer and Hughes 2012).

### Acquisition of Transcriptome Profiles and DEGs

A single colony of each homozygous knockout was selected and inoculated into 4 mL YPD at 30 °C and shaken to saturation. The saturated culture was then restored to OD660 = 0.16 in 4 mL YPD for 3 to 4 h until the OD660 reached 0.65 to 0.75. Total RNA was then extracted from cell lysates using the RNeasy Mini Kit (Qiagen) according to the manufacturer’s instructions. The quality of RNA was determined by the A260/A230 ratio, A260/A280 ratio, and concentration on a NanoDrop spectrophotometer (Thermo Scientific). RNAs with an A260/A230 ratio > 2 and an A260/A280 ratio in the range of 1.8 to 2.2 were considered acceptable and used in subsequent experiments. RNA extracts were obtained from at least 2 biological replicates for each homozygous knockout.

Library construction was performed using 1 μg of total RNA in each sample according to the guidelines of the VAHTSTM mRNA-seq V3 Illumina Library Preparation Kit (Vazyme). A Library with a concentration of more than 2 ng/μL measured by Qubit (Thermo Scientific) and average fragment lengths between 400 and 450 bp measured by Qsep1 (BiOptic) was subsequently sequenced on a HiSeq system (Illumina) to obtain ∼2.0 Gbp of 150 bp paired-end sequencing reads.

To estimate the RNA abundance in each homozygous knockout, we mapped the adaptor-trimmed and quality-filtered (Bolger et al. 2014) reads to the reference genome by STAR (Dobin et al. 2013). Then, transcripts per million (TPM) read values (Wagner et al. 2012) were estimated based on the mapping results after removing batch effects by the R package limma (Ritchie et al. 2015). All software listed above was used with default settings. Genes in the uracil biosynthesis pathway and genes with low expression levels in the wild-type strain (TPM < 1) were excluded, and a gene set including 5,359 verified genes in both BY and RM was obtained for further analysis.

To identify the DEGs in knockout strains compared with wild-type strains, we used the negative binomial generalized linear models provided by the software package DESeq2 (Love et al. 2014). Additionally, we used the apeglm method for effect size shrinkage to remove noise and preserve significant differences (Zhu et al. 2019). Finally, compared with the corresponding wild-type strain, genes in the homozygous knockouts meeting the following conditions were considered DEGs: the expression changed (up- or downregulated) more than 1.2-fold, and the adjusted *P* value was less than 0.01 (*P*_adj_ < 0.01). We occasionally used other thresholds, which were stated accordingly in the text.

### Calculation of the Pleiotropy Value of Knockout Effects on Gene Expression

The mean value of log_2_TPM in replicates of the wild-type strain or knockouts was taken as the expression level of each gene. The coexpression networks of 5,359 genes were analyzed by WGCNA (Langfelder and Horvath 2008) with the following parameters unless otherwise specified: minModuleSize = 10 and mergeCutHeight = 0.1. Genes were then classified into different coexpressed modules, and the unassigned genes were not considered in subsequent analyses. The number of coexpressed modules of DEGs was used to estimate the pleiotropic effects of the knockouts on gene expression to eliminate the redundancy of the coexpressed genes.

### Gene Feature Analysis

The activators and repressors among 100 TFs were manually confirmed according to their annotations from the SGD (Cherry et al. 1998). The direct targets of 100 TFs were retrieved from YEASTRACT (Teixeira et al. 2023) by the function of “Search for target genes” with the documented parameter “DNA binding and expression evidence.” The GO analysis of DEGs for each knockout was performed with the R package “cluster Profiler” under default settings (Yu et al. 2012). We identified enriched GO terms based on criteria employed in a previous study (Liu et al. 2020), in which the fold enrichment was greater than 2 and the *P* value was less than 0.05.

### Acquisition of Morphological Data and Altered Traits

The morphological data were measured in the wild-type strain and homozygous knockouts according to previous studies with slight modifications (Ohya et al. 2005; Okada et al. 2015). The cell culture method used here was the same as that described in the “Acquisition of Transcriptome Profiles and DEGs” section. Fifteen microliters of the log-phase culture (OD660 = 0.65 to 0.75) was transferred to a 96-well plate and mixed with 85 μL of YPD, 12.5 μL of 37% formaldehyde solution, and 12.5 μL of 1 M potassium phosphate buffer. After shaking the plate at 150 rpm and 25 °C for 1 h, the cells were then fixed with 10 μL of 37% formaldehyde solution, 10 μL of 1 M potassium phosphate buffer, and 80 μL of ddH_2_O for 45 min at room temperature. Cell walls were stained with FITC-ConA (concanavalin A conjugated with fluorescein isothiocyanate). Cell nuclei were stained with Hoechst. We did not stain actin because rhodamine phalloidin dye is not stable enough to support the subsequent high-throughput automatic image capture. Images of stained cells were then captured with an IN Cell Analyzer 2,500 (GE Healthcare) using a 100× objective lens. For each strain, 3 biological replicates were performed. For each biological replicate, 3 technical replicates of staining and image capture were performed. In each batch, the corresponding wild-type strain was added as a control.

By analyzing the images using CalMorph under default settings (Negishi et al. 2009), 187 morphological traits were obtained for each cell. Three traits (D161_A1B, D165_A1B, an D172_A1B) with a cell number < 300 were excluded from further analysis. We removed batch effects with the R package “LIMMA” (Ritchie et al. 2015) to obtain the refined values of the morphological traits. To identify significantly altered traits, each trait in all biological and technical replicates of a specific knockout and the wild-type strain in the same batch was fit using a linear mixed-effects model with or without the mutation effects, and its significance was then obtained by comparing the 2 models with ANOVA. The significantly altered traits were defined using the cutoffs *P* < 0.01 and FC > 1, with FC being the fold change of the average value of each trait in the TF knockout relative to that in the wild-type strain. We occasionally used other thresholds, which were stated accordingly in the text.

### Quantification of Pleiotropic Effects of Knockouts on Cell Morphological Traits

Individual observations were quantile-normalized across morphological traits and then averaged for each of the 100 × 184 TF-trait combinations. The reproducibility of each TF knockout was assessed by the Pearson's *R* of the FC for 184 morphological traits in biological replicate of 100 TF knockouts compared to wild-type strains. Based on the 100 averaged values of each trait, the 184 traits were clustered using the R package “apcluster” (negDistMat, *r* = 2) (Bodenhofer et al. 2011). The number of trait clusters containing any significantly altered trait was taken as the pleiotropy value of the knockout effects on cell morphological traits.

### Definition of the Allele States of QTLs

The eQTLs for each gene and the values of variance explained by each eQTL were obtained from Albert et al.'s study (supplementary table S10, Supplementary Material online) (Albert et al. 2018). The updated version of genotypes for ∼1,011 segregants was downloaded from Peter et al.'s study (Peter et al. 2018). Benefiting from Peter et al.'s study (Peter et al. 2018), the Chinese lineage included the BAL, BAH, BAG, BAM, and BAQ strains, and the Taiwanese lineage included the CEG, CEI, and AMH strains. We then defined the ADT and SACE_GAV strains as the BY lineage according to the minimum SNP number compared to the S288c strain. Similarly, the BSE and CFS strains were defined as the RM lineage because they have the minimum SNP number compared to the RM11-1A strain. In addition to these strains, 21 other strains were randomly sampled from other clades (supplementary table S11, Supplementary Material online). The allele states for each QTL in the above 33 strains were selected according to their chromosomal locations.

For the allele states of the outgroup species SP, we only focused on the coding regions of each gene. The coding sequences of SP strain N17 and SC strain S288c were downloaded from the SGD (Cherry et al. 1998). The allele states of SP were determined based on alignments of orthologous coding sequences in SP and S288c.

## Supplementary Material

msae189_Supplementary_Data

## Data Availability

All raw data from Illumina sequencing have been deposited in NCBI BioProjects under accession number PRJNA1039971.
