# Supplementary material for: Most Pleiotropic Effects of Gene Knockouts Are Evolutionarily Transient in Yeasts
Source: Mol Biol Evol. 2024 Sep 6;41(9):msae189. doi: 10.1093/molbev/msae189 (PMC11414406; doi:10.1093/molbev/msae189)
Supplement: msae189_Supplementary_Data [file msae189_supplementary_data.zip › Supplemetary_figures_v10.pdf]

Supplementary figures

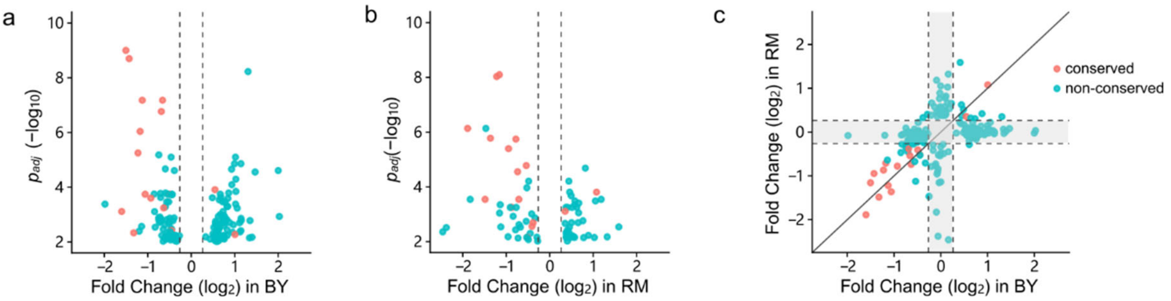

Fig. S1. An example of the DEGs analysis performed by *CRZ1Δ*. (a) The standard volcano plot in BY. (b) The standard volcano plot in RM. (c) The comparison of fold changes between BY and RM for DEGs. The DEGs under the same cutoff conditions (padj<0.01&| fc|>1.2) are outside the gray area and conservative DEGs are marked with red. A black line indicates that Y = X.

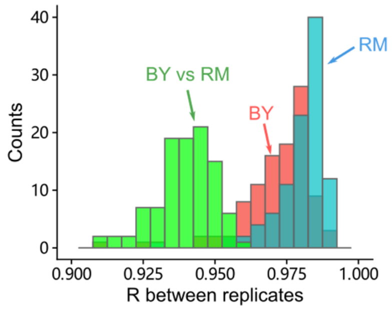

Fig. S2. The distribution of Pearson's R of expression profiles between two biological replicates for knockouts of 100 TFs and the wild-type strain in RM, BY and cross-strains.

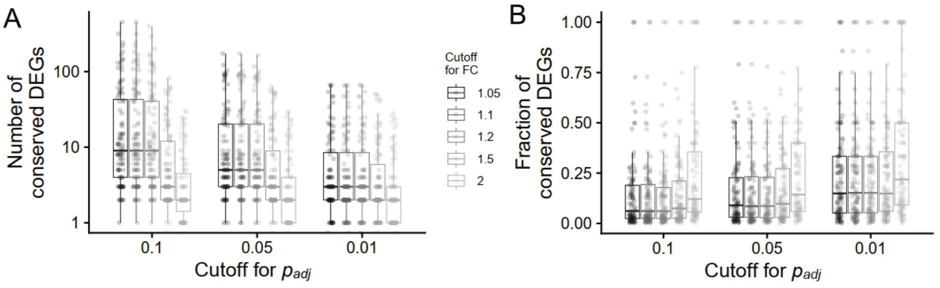

Fig. S3. The number (A) and the fraction (B) of conserved DEGs of 100 TF knockouts under different parameters to define DEGs.

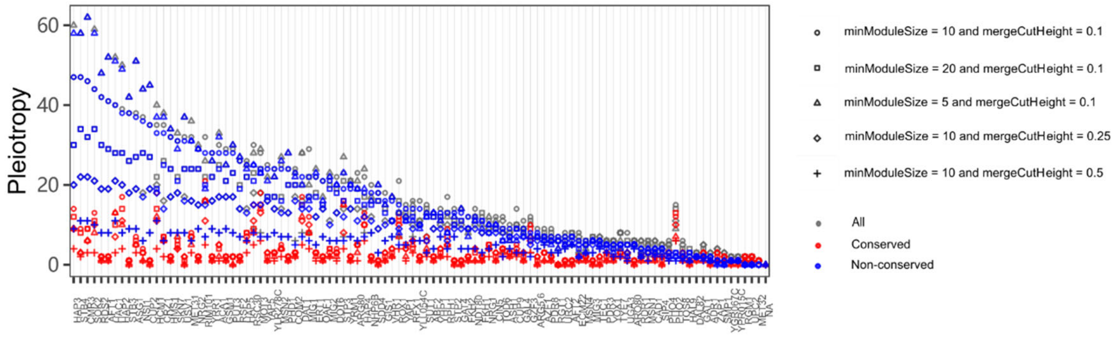

Fig. S4. The pleiotropic effects of 100 TF knockouts under different parameters to define coexpressed modules.

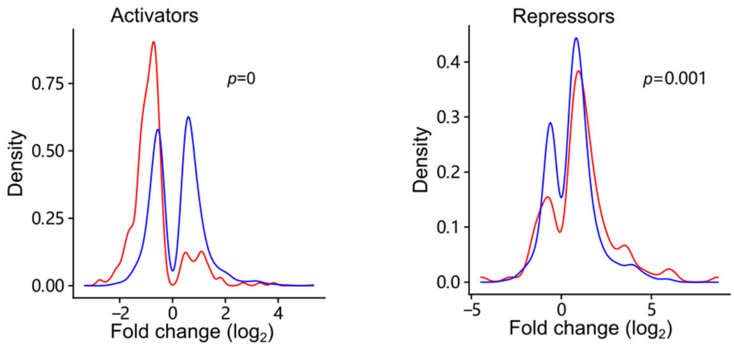

Fig. S5. The density curves of log_2FC for conserved and non-conserved effects in 42 activators and 13 repressors excluding the effects on the genes *RIB4*, *MDH2* and *AAP1*. The *p* values obtained from the Kolmogorov-Smirnov test between each of the two curves are labeled.

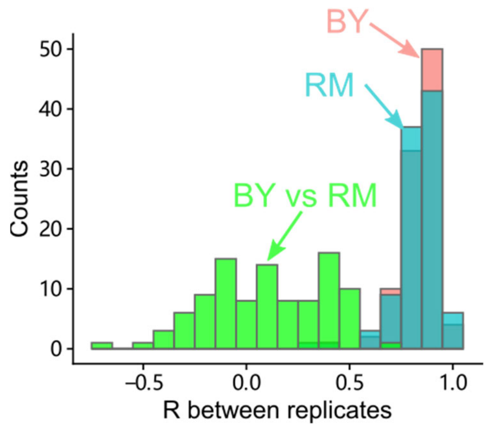

Fig. S6. The distribution of Pearson's R of the fold change for 184 morphological traits in BY, RM, and cross-strains compared to wild-type strains.

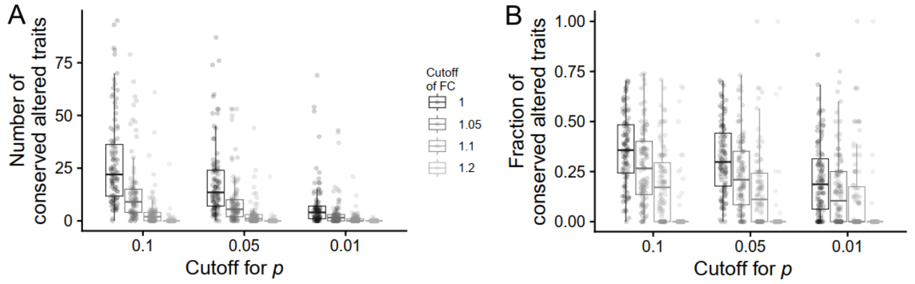

Fig. S7. The number (A) and the fraction (B) of conserved traits of 100 TF knockouts under different parameters to define altered traits.

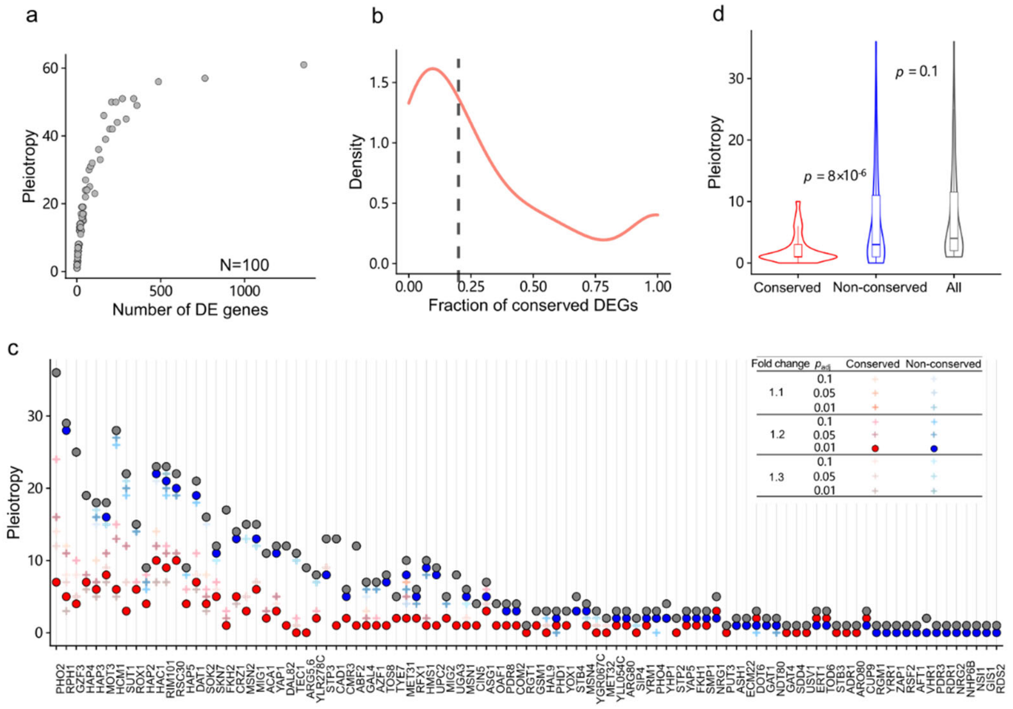

Fig. S8. The knockout effects of 100 TFs on gene expression in RM. The DEGs for each TF knockout were derived using the same procedure as in BY. (a) The number of DEGs and corresponding pleiotropic effects estimated by the coexpressed modules across 100 TF knockouts. (b) The density curve of the fraction of conserved DEGs across 100 TF knockouts. The dashed line indicates the median value. (c) The pleiotropic effects of 100 TF knockouts were categorized into conserved or non-conserved via a comparison with the DEGs identified in BY. Conserved pleiotropic effects are colored red, non-conserved pleiotropic effects are colored blue, and overall pleiotropic effects are colored gray. Specific thresholds used for DEG identification in BY are listed in the top-right corner. Fold change indicates both up or down-regulation. (d) The comparison of conserved, non-conserved, and overall pleiotropic effects. The p values obtained from the Wilcoxon test between each pair of groups are labeled.

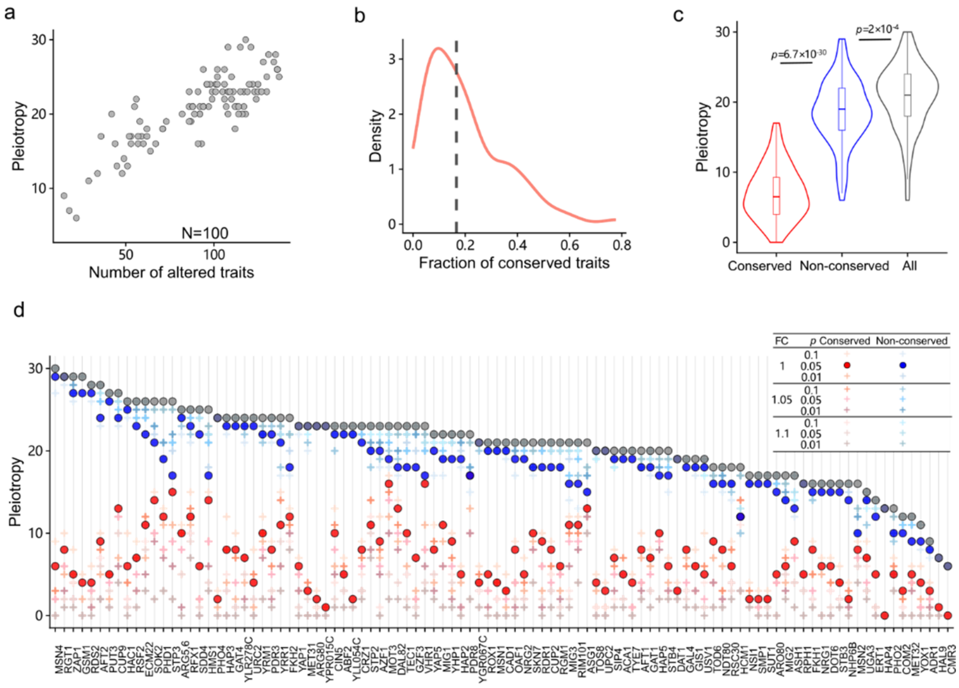

Fig. S9. The knockout effects of 100 TFs on cell morphology in RM. The significantly traits for each TF knockout were derived using the same procedure used as in BY. (a) The number of altered traits and corresponding pleiotropic effects estimated by the morphological trait clusters across 100 TF knockouts. (b) The density curve of the fraction of conserved traits across 100 TF knockouts. The dashed line indicates the median value. (c) The pleiotropic effects of 100 TF knockouts were categorized into conserved or non-conserved via a comparison with the significantly altered traits identified in BY. Conserved pleiotropic effects are colored red, non-conserved pleiotropic effects are colored blue, and overall pleiotropic effects are colored gray. Specific thresholds used for significantly altered traits identification in BY are listed in the top-right corner. Fold change indicates both up or down-regulation. (d) The comparison of conserved, non-conserved, and overall pleiotropic effects. The *p* values obtained from the Wilcoxon test between each pair of groups are labeled.
